# COMPARING THE PROGNOSTIC VALUE OF GERIATRIC HEALTH INDICATORS: A POPULATION-BASED STUDY

**DOI:** 10.1093/geroni/igz038.2269

**Published:** 2019-11-08

**Authors:** Alberto Zucchelli, Davide Vetrano, Giulia Grande, Amaia Calderon-Larranaga, Laura Fratiglioni, Alessandra Marengoni, Debora Rizzuto

**Affiliations:** 1 Department of Clinical and Experimental Sciences, University of Brescia, Brescia, Italy; 2 Aging Research Center, Department of Neurobiology, Care Sciences and Society, Karolinska Institutet and Stockholm University, Stockholm, Sweden

## Abstract

Several indicators associated with poor outcomes in older persons have been developed, but a direct comparison of their accuracy is lacking. Knowing which indicator performs better in the prediction of specific outcomes could help health care providers to choose the most suitable one. We compared the accuracy in predicting different clinically-relevant outcomes of five indicators: frailty index (FI), frailty phenotype (FP), the Health Assessment Tool (HAT), walking speed (WS), and multimorbidity. Data from the Swedish National Study on Aging and Care in Kungsholmen, an ongoing population-based study including 3363 people 60+, were used. The ability of the five indicators to predict mortality (3- and 5-year), unplanned hospitalizations (1- and 3-year), and 2+ health provider contacts (6 months prior and after assessment) was compared using the area under the ROC curves (AUC). FI, WS, and HAT showed the best accuracy in the prediction of mortality (AUC for 3-year mortality: 0.84, 0.85, 0.87 respectively; AUC for 5-year mortality: 0.84, 0.85, 0.86 respectively; all p < 0.05). Unplanned hospitalizations were better predicted by the FI (AUC: 1-year 0.73; 3-year 0.72) and HAT (AUC: 1-year 0.73; 3-year 0.71).The most accurate predictor of multiple contacts with health providers was multimorbidity (AUC: 0.67; p < 0.05). All indicators, but multimorbidity, showed higher accuracy among older individuals (75+ years). Different indicators can be used to support physicians during their decision-making process. Some of these tools may also be used to forecast future use of health-care resources, including both hospital-based services and outpatient ones .

